# Isotemporal Associations of Patterns and Domains of Sedentary Behavior and Physical Activity with Sleep Quality in Pregnant Women in Saudi Arabia

**DOI:** 10.3390/healthcare13192397

**Published:** 2025-09-23

**Authors:** Abdullah Bandar Alansare, Ghareeb Omar Alshuwaier, Nada Khojah, Saja Abdullah Alghamdi, Alawyah Alsalman, Om Kalthom Sowadi, Hadeel Saad, Bethany Barone Gibbs

**Affiliations:** 1 Department of Exercise Physiology, College of Sport Sciences and Physical Activity, King Saud University, Riyadh 11451, Saudi Arabia; galshuwaier@ksu.edu.sa (G.O.A.); nkhojah@ksu.edu.sa (N.K.); sajaalghamdi19@gmail.com (S.A.A.); omkalthom.sowadi@hotmail.com (O.K.S.); 443203529@student.ksu.edu.sa (H.S.); 2 Department of Physical Education, College of Sport Sciences and Physical Activity, King Saud University, Riyadh 11451, Saudi Arabia; alwyah2020@gmail.com; 3 Department of Epidemiology and Biostatistics, School of Public Health, West Virginia University, Morgantown, WV 26506, USA; bethany.gibbs@hsc.wvu.edu

**Keywords:** pregnancy, maternal, midwifery, sleep period, activity domains, activity patterns

## Abstract

**Background:** Although higher sedentary behavior (SB) and lower physical activity (PA) have been associated with poor prenatal sleep quality, the influence of specific exchanges of SB and types of PA on sleep quality during pregnancy remains unexplored. **Objectives:** This secondary, cross-sectional analysis examined associations between the statistical replacement of patterns (weekdays, weekends) and domains (leisure, occupational, commuting) of SB with moderate (MPA), vigorous (VPA), or moderate-to-vigorous (MVPA) PA and sleep quality among pregnant women in Saudi Arabia. **Methods:** Participants (n = 935; age = 30 ± 5.6 years; first trimester = 24.1%, second trimester = 33.9%, third trimester = 42.0%) self-reported their patterns and domains of SB, PA, and sleep quality using validated instruments. Adjusted isotemporal substitution models evaluated the associations of exchanging 30 min of different SB and PA with sleep quality. **Results:** Replacing 30 min of total or leisure SB on weekdays with 30 min of MPA was associated with improved sleep quality (β = −0.519 and −0.590, respectively; *p* < 0.05) only among those in their first trimester. Paradoxically, replacing 30 min of total, leisure, occupational, or commuting SB across the week, on weekdays, and weekends with 30 min of VPA was associated with poor prenatal sleep quality (β ranged between 1.258 and 7.217; *p* < 0.05 for all). Exchanging SB with MPVA or different domain-specific SB did not influence sleep quality (*p* > 0.05 for all). **Conclusions:** These novel findings suggest that although replacing SB with PA may help enhance sleep quality in pregnant women, particularly during the first trimester, the underlying associations are likely multifaceted. The variable relationships observed emphasize the importance of considering patterns and domains of SB and intensity of PA used as a replacement, rather than total duration solely, to improve prenatal sleep quality, especially during early pregnancy, particularly in Saudi Arabia.

## 1. Introduction

Poor sleep quality is a significant challenge that most pregnant women endure. A meta-analysis, which included more than 11,000 pregnant women, revealed that the prevalence of poor sleep quality during the first, second, and third trimesters was 54%, 49%, and 70%, respectively [1]. This prevalence also extends into the postpartum period, where 71% of women reported poor sleep quality during the early months after delivery [2]. Importantly, poor prenatal sleep quality significantly contributes to pregnancy complications (e.g., gestational diabetes), postpartum disorders (e.g., postpartum depression), and infant issues (e.g., increased body mass index, poorer offspring sleep and development) [3,4,5]. These ramifications have urged health and scientific communities, as well as researchers, to examine the potential modifiable risk factors that exacerbate poor sleep quality during pregnancy [6,7] and explore the effectiveness of various interventions to boost sleep quality among pregnant women [8,9].

Sedentary behavior (SB) (i.e., any waking behavior that occurs while seated, lying, or reclining with limited energy expenditure) [10] and physical inactivity (i.e., not complying with 150 min/week of moderate physical activity [MPA]) [11] have been proposed as modifiable risk factors for poor sleep quality during pregnancy [6,7]. Indeed, SB and physical inactivity are highly prevalent among pregnant women worldwide. A previous systematic review, which included 26 studies from 11 countries, revealed that pregnant women devoted the majority (>50%) of their waking time in SB [12], with a modest amount (~20%) of this time spent on prolonged bouts (i.e., ≥30 min) [13]. Alongside this, emerging research suggests that more than half of pregnant women do not meet MPA recommendations [14,15]. As such, strategies that target reducing SB and promoting MPA may be essential and impactful for improving prenatal sleep quality.

Of particular interest, the domains (i.e., leisure, occupational, or commuting) and patterns (i.e., weekends or weekdays) of SB appear to correlate differently with sleep quality during pregnancy. For example, a recent study revealed that higher total and leisure SB, especially on weekends, were associated with a higher risk of poor sleep quality in pregnant women [7]. Yet, no comparable relationships were observed for the other domains or patterns of SB. Hence, exploring strategic approaches to reduce leisure and weekend SB may be a more promising strategy for enhancing sleep quality and overall pregnancy health. Specifically, exchanging time spent in SB with physical activity (PA) may yield the most beneficial influence on prenatal sleep quality. Nonetheless, this proposed strategy has not been previously explored. Isotemporal substitution analysis is a statistical technique that can estimate the hypothetical effect of replacing time spent in one behavior with another [16], such as whether decreasing certain domains and patterns of SB by increasing certain types of PA would be associated with better sleep quality during pregnancy.

Therefore, the main aims of this secondary analysis were to (1) examine the associations of statistically replacing total, leisure, occupational, and commuting SB with MPA, vigorous (VPA), or moderate-to-vigorous (MVPA) PA and sleep quality in pregnant women by trimester and (2) separately evaluate these relationships on weekend days and weekdays. The associations of exchanging different domains of SB with sleep quality were also explored. It was hypothesized that statistically substituting SB with PA would be associated with improved sleep quality among pregnant women. It was hypothesized that these associations would be more apparent when replacing total and leisure SB with PA or other types of SB, and on weekend days as compared to weekdays.

## 2. Materials and Methods

This study was a secondary analysis of a previously published cross-sectional investigation that evaluated 24-hour movement behaviors during pregnancy [14]. The data collection was completed between 3 July 2023 and 24 August 2023, at obstetrics and gynecological clinical centers across Saudi Arabia. The study’s personnel were stationed in the waiting areas at private and public obstetrics and gynecology clinics. They were assigned to recruit and administer questionnaires to all pregnant women visiting these clinics who were permanent residents of Saudi Arabia. The initial sample size for the original study was 952 pregnant women [14]. Of them, 17 participants were excluded due to unreasonable total SB (n = 2), sleep (n = 2), and PA (n = 11) or missing body weight or height (n = 1). Therefore, the total number of the included pregnant women in the analytic sample of the current analysis was n = 935.

The original study was approved by the Institutional Review Board at King Saud University (No: KSU-HE-23-516) and was performed in line with the Declaration of Helsinki. The Strengthening the Reporting of Observational Studies in Epidemiology (STROBE) guidelines were utilized to report this manuscript.

### 2.1. Measurements

#### 2.1.1. Participants’ Demographics and Health-Related Measures

Pregnant women self-reported their demographics and health-related measures (Table 1). The average age of the included pregnant women was 30.0 ± 5.6 years. These participants were fairly evenly distributed across the three trimesters and tended to be housewives (76.8%), with at least an undergraduate degree (58.7%), and a previous child (61.9%). The average reported times spent in SB, MPA, VPA, and MVPA were 6.8 h/day, 30.0 min/day, 2.1 min/day, and 32.1 min/day, respectively. Furthermore, Supplemental Table S1 presents descriptive comparisons of demographics and health-related measures by trimester. Pregnant women across the first, second, and third trimesters tended to have comparable age, educational, occupational, health, and smoking statuses, SB, and sleep quality. Yet, the percentage of pregnant women who reported having children appeared to be higher among those in their third trimester compared to first or second trimester (69.5% vs. 56.9% and 56.2%, respectively). Moreover, pregnant women in their first trimester tended to engage in less MPA compared to those in their second or third trimester (22.5 min/day vs. 32.4 min/day and 32.5 min/day, respectively). This latter difference was also reflected in MVPA (min/day).

#### 2.1.2. Sleep Quality Evaluation

Sleep quality was assessed using the Arabic version of the Pittsburgh Sleep Quality Index (PSQI) [17]. This questionnaire is one of the widely used tools to evaluate sleep quality [18], and its validity and reliability in pregnant women have been previously established [19]. This questionnaire consists of 9 items summed into 7 different sleep quality components, including daytime dysfunction, sleep duration, subjective sleep quality, sleep disturbance, sleep efficiency, sleep latency, and the use of sleep medication. The score for each component can vary from 0 to 3 points. The sum of all components was used to estimate the global PSQI, which falls between 0 and 21 points. Then, the global PSQI was utilized as a continuous outcome to assess sleep quality, where a higher score indicates worse sleep quality [20].

#### 2.1.3. Sedentary Behavior Patterns and Domains Evaluation

The Arabic version of the Sedentary Behavior Questionnaire (SBQ) was utilized to estimate times spent in total and domain-specific (i.e., leisure, occupational, and commuting) SB (hours/day) on a weekday or weekend day [21]. The validity and reliability of this questionnaire to measure SB in pregnant women have also been confirmed [22]. The questionnaire includes 9 distinct repeated items for weekdays and weekend days, designed to assess various SB domains. To elaborate, 7 of these items (i.e., playing a musical instrument, sitting and listening to music, playing computer or video games, sitting and reading a book or magazine, doing artwork or crafts, watching TV, and sitting and talking on the phone) estimate the time spent in leisure SB. On the other hand, the other two items estimate times spent in occupational (i.e., doing paperwork or computer work) or commuting (i.e., sitting and driving a car, bus, or train) SB [23].

Total SB on weekdays or weekend days was calculated by summing the times spent on all items of SB reported on weekdays and weekend days separately. Thereafter, the total SB per day was estimated using the following standardized formula: total SB per day = ([total SB on a weekday × 5] + [total SB on a weekend day × 2])/7. Times spent in leisure, occupational, and commuting SB averaged across the week were similarly computed. Times spent in each domain of SB on weekdays, weekend days, and averaged across the week were calculated separately and using similar formulas [24].

#### 2.1.4. Moderate and Vigorous Physical Activity Evaluation

The times spent in MPA and VPA were estimated using the Arabic short version of the International Physical Activity Questionnaire (IPAQ) [25]. Similar to other questionnaires, the validity and reliability of the IPAQ to measure MPA and VPA in pregnant women have been previously established [26]. This questionnaire contains four specific questions about the number of days and min per day individuals performed MPA or VPA for at least 10 min at a time during the past 7 days. These estimates were used to compute the average min/day spent in MPA, VPA, and MVPA.

### 2.2. Statistical Analyses

Participants’ demographics and health-related measures were reported as mean ± standard deviation or frequency and percentage, as appropriate. Adjusted isotemporal substitution models were fitted to examine the influence of substituting SB with MPA or VPA on sleep quality, while holding the total activity time (i.e., the sum of total SB, MPA, and VPA) constant and adjusting for covariates (i.e., age, smoking status, having children, education, occupation, and chronic disease status). Further adjusted isotemporal substitution models were fitted to evaluate the influence of exchanging domain-specific SB with sleep quality while holding the total activity time constant and controlling for covariates. To facilitate the interpretations of the findings, all SB, MPA, VPA, MVPA, and total activity time were rescaled into 30 min/day prior to entering them into the regression models. After this, the β observed represents the hypothetical effects of replacing 30 min of one activity behavior with 30 min of another activity behavior, while adjusting for covariates and keeping total time in all other activities constant. The normality assumption of the model residuals was checked and confirmed for all models. All analyses were completed using JASP software (JASP 0.15 Version). The significance level for all analyses was set at *p*-value < 0.05.

## 3. Results

Table 2 displays the influence of exchanging total SB and PA on sleep quality in pregnant women. No significant associations were found when replacing 30 min of total SB across the week or on weekend days with 30 min of MPA; however, replacing 30 min of total SB on weekdays with 30 min of MPA was associated with improved sleep quality (β = −0.519; *p* < 0.05) only among pregnant women in their first trimester. Paradoxically, replacing 30 min of total SB across the week, on weekdays, and weekend days with 30 min of VPA was associated with reduced sleep quality among the overall sample (β = 2.473, 2.459, and 2.512, respectively; *p* < 0.05 for all). Once the trimester was considered, these detrimental associations were of greater magnitude in the first trimester (β = 6.875, 6.758, and 6.944, respectively; *p* < 0.05 for all) and seemed to attenuate with increasing trimester, though they remained statistically significant. Substituting 30 min of total SB across the week, on weekdays, and weekend days with 30 min of MVPA did not significantly influence prenatal sleep quality.

Partitioning total SB into domains yielded similar significant associations with sleep quality for leisure SB (Table 3). However, the favorable association with sleep quality was attenuated and became non-significant when 30 min of occupational or commuting SB on weekdays was replaced with 30 min of MPA (Supplemental Tables S2 and S3).

Furthermore, the influence of substituting times spent in domain-specific SB on sleep quality in pregnant women was reported in Table 4. These analyses suggest that exchanging times between domains of SB was not associated with sleep quality in pregnant women (*p* > 0.05 for both).

## 4. Discussion

**Figure 1:**
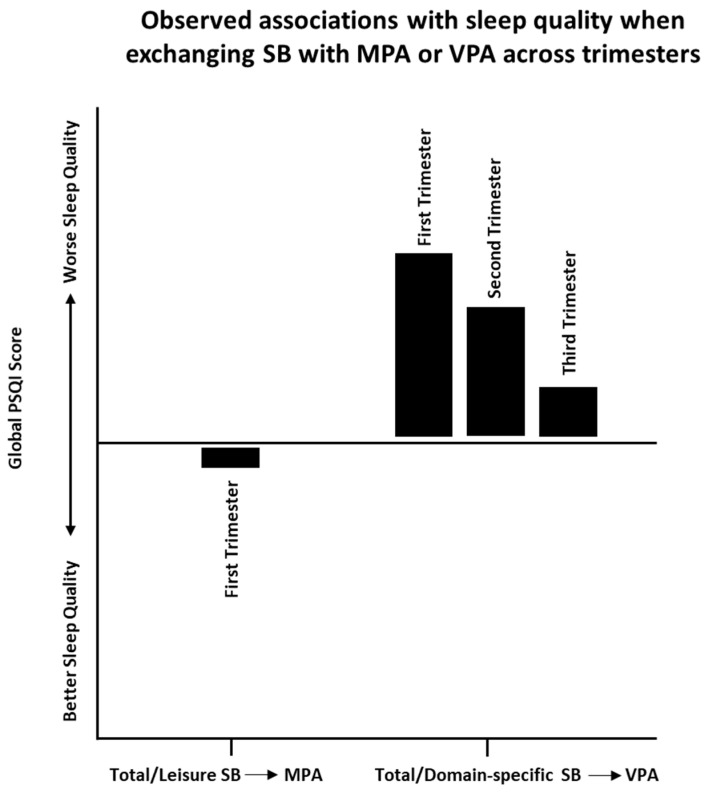
Summary of key substitution influence across trimesters. Substituting total or leisure SB with MPA was significantly associated with more optimal prenatal sleep quality in pregnant women in their first trimester. Substituting total or domain-specific (leisure, occupational, commuting) SB with VPA was significantly associated with poorer prenatal sleep quality in pregnant women in their first, second, or third trimester.

To our knowledge, this analysis is the first to investigate whether statistically reallocating SB to PA is a potential strategy for promoting sleep health during pregnancy. The primary findings are summarized in Figure 1. Consistent with the broader sleep health literature, substituting total or leisure SB on weekdays with the same duration of MPA was associated with favorable maternal sleep quality, specifically in pregnant women in their first trimester. Paradoxically, statistically replacing total or domain-specific SB with an equivalent amount of VPA was associated with poor prenatal sleep quality, especially during early pregnancy. Exchanging SB with MPVA or different domain-specific SB did not emerge as a promising strategy to enhance sleep quality during pregnancy. These novel findings indicate that the influence of replacing patterns and domain-specific SB with different PA on prenatal sleep quality is complex. The paradoxical relationships observed highlight the importance of considering patterns and domains of SB and intensity of PA used as a replacement, rather than total duration solely, to improve maternal sleep quality.

Research evaluating the effects of statistically reallocating SB to PA on sleep quality in adults is scarce. Among the existing studies, none included pregnant women, and the overall findings are inconsistent and remain inconclusive. For example, while studies found that replacing total SB with an equal duration of LPA, MPA, or VPA improved sleep quality among young or older adults [27,28], others reported no influence on sleep quality when total SB was substituted with LPA or MVPA in middle-aged or older adults [27,29]. Herein, the present investigation further conferred novel paradoxical findings and revealed that replacing total or leisure SB with MPA was associated with improved sleep quality, particularly during early pregnancy. In parallel, reallocating total or domain-specific (i.e., leisure, occupational, or commuting) SB to a comparable duration of VPA was associated with poor prenatal sleep quality; the substitution with MVPA did not appear to influence sleep quality. Considering the complex and contradictory influence of statistically displacing SB with different intensities of PA on sleep quality, it is challenging to determine the superior PA substitute for SB to improve sleep quality. Nonetheless, these data highlight the potential benefits of reallocating SB to PA for better sleep quality.

Although consensus has yet to be reached, the adverse impacts on prenatal sleep quality observed when SB was replaced with VPA align with some existing evidence in non-pregnant adults. For instance, a recent large multinational cohort study of physically active adults (n = 14,689) found that greater evening VPA disrupted the subsequent sleep and nocturnal autonomic regulation [30]. A randomized crossover experiment also reported impaired sleep quality and nocturnal autonomic function, increased body temperature, and muscle damage after nighttime VPA in athletes [31]. By contrast, the favorable associations detected when SB was substituted with MPA also conform to findings documented in adults. Accumulating research demonstrates that light PA (LPA) or MPA interventions improve sleep quality in pregnant women [32] and recreational runners [33]. This body of evidence suggests that the timing and intensity of PA substitute for SB may be complex underlying determinants of how sleep quality effects manifest.

To elaborate, VPA, especially when performed in the evening and close to bedtime, but not MPA, may exaggerate the body’s physiological responses by increasing nocturnal sympathetic activity, heart rate, core body temperature, muscle damage, or diminishing parasympathetic activity [31]. These deleterious changes may increase sleep latency, delay sleep onset, decrease sleep duration, aggravate sleep disturbance and awakenings after sleep onset, and/or reduce sleep efficiency [34], leading to poor sleep quality. As people living in a hot environment tend to delay their PA into cooler nighttime [35], pregnant women in the present study would have been likely to perform their VPA in the evening. This is further supported by a recent cross-sectional report, which observed that just over a quarter of Saudi women perform PA in the evening [36]. Nonetheless, further research exploring this hypothesis is warranted to achieve more conclusive evidence regarding the influence of exchanging SB with MPA or VPA on prenatal sleep quality.

Notably, the current study provided an early, favorable indication suggesting a beneficial influence on maternal sleep quality when SB was substituted with an equal amount of MPA only in pregnant women in their first trimester; pregnant women in their second or third trimester showed a trend toward improvement in sleep quality, but the change was not statistically significant. A previous systematic review and meta-analysis of randomized controlled trials showed that PA interventions, including MPA, improved prenatal sleep quality, regardless of gestational age [32]. It is hypothesized that factors unique to the first vs. the second or third trimester may explain these discrepancies. For example, during early pregnancy, cardiac output and tidal volume increase, resulting in a greater ability to engage in MPA [37]; however, as pregnancy progresses to the second and third trimester, these cardio-respiratory functions decline, potentially lowering PA tolerance [38]. In addition, pregnant women in later pregnancy stages are likely to experience discomfort [39]. These unfavorable changes could have limited the beneficial influence of MPA among pregnant women in their second or third trimester in the present study [40].

Furthermore, the favorable influence on sleep quality detected appeared to be largely driven by the replacement of leisure rather than occupational or commuting SB on weekdays with MPA. Two primary factors may explain this finding. First, the utilized SB questionnaire (i.e., SBQ) captured several types of leisure SB (e.g., TV viewing, reading a book), while it only encapsulated one type of occupational (i.e., doing paperwork or computer work) and commuting (i.e., sitting and driving a car, bus, or train) SB [24]. Measuring several types of SB usually allows for providing more accurate and reliable estimates of the associations than a single-type assessment [41]. Secondly, leisure SB on weekdays is often characterized by a substantial amount of TV viewing [42], usually occurring at night before sleep [43], tending to delay sleep–wake time [44], and worsening sleep quality compared to other domain-specific SB in pregnant and non-pregnant adults [7,45]. Notably, the current study also demonstrated that replacing this leisure SB with other domain-specific SB did not alleviate existing poor sleep quality in pregnant women. Therefore, movement behavioral interventions aiming to improve maternal sleep health should not only target increasing MPA but also limit leisure SB by substituting it with MPA.

### 4.1. Strengths and Limitations

This study demonstrated key strengths that are worth mentioning. First, the large, recruited sample consisted of women from different stages of pregnancy and diverse Saudi cities, improving the generalizability of the findings. Moreover, the utilization of isotemporal substitution analyses is another strength as it allows the examination of real-life behavioral trades (i.e., reallocating 30 min from SB to PA) and contrasting the effects of different PA intensities (i.e., MPA vs. VPA) [16]. This consideration facilitates the interpretation of the results and provides more realistic directions to the public and decision-makers compared to conventional analyses. Another strength was assessing the reallocations of SB with PA while considering the domains (i.e., leisure, occupational, or commuting) and patterns (i.e., weekends or weekdays) of SB. This was of particular significance, as previous studies had revealed varying associations of distinct domains and patterns of SB with health outcomes, including sleep health during pregnancy [7,46,47]. Still, caution is warranted when interpreting the current findings because SB, PA, and sleep quality were self-reported. Such assessment tools are susceptible to recall bias and/or inaccurate estimations [48]. As such, the results may differ if more accurate and reliable instruments such as accelerometers were utilized. Another important limitation is that IPAQ lacks sufficient granularity for LPA assessment, which may be a more feasible and beneficial substitution, particularly in later trimesters, given fatigue and mobility constraints. Moreover, SBQ cannot distinguish between short vs. long bouts of SB, failing to examine the influence of replacing different bouts of SB with PA on sleep quality in pregnant women.

### 4.2. Clinical Implications

The results of this study suggest that replacing SB with MPA can improve prenatal sleep quality, particularly during early pregnancy. These findings encourage the amalgamation of the movement behavior substitution into strategies, interventions, and recommendations aimed at enhancing sleep health during pregnancy. As pregnant women are generally recommended to accumulate at least 150 min/week of MPA to reduce the risks of pregnancy complications [11], the present study further complements these recommendations by suggesting the reallocation of SB to MPA for more optimal maternal sleep health. For example, the current results suggest that replacing 30 min of TV viewing on weekdays with walking may improve prenatal sleep quality during early pregnancy. Through the adoption of this strategy, pregnant women, midwives, and obstetricians may not only reduce the risks of adverse pregnancy outcomes but also boost holistic health during pregnancy. Still, further longitudinal studies and randomized controlled trials are needed to confirm the effects observed in this hypothetical movement behavior exchange study. Future research should also explore the interaction terms between employment status and SB domains (e.g., occupational SB), which might reveal more nuanced associations with prenatal sleep health.

## 5. Conclusions

In summary, this investigation uniquely explored the potential influence of reallocating SB to PA on sleep quality among pregnant women in Saudi Arabia. Exchanging total or leisure SB on weekdays with an equivalent amount of MPA was associated with improved sleep quality in pregnant women, specifically during early pregnancy. Conversely, equally displacing total and domain-specific SB with VPA was associated with poor sleep quality during pregnancy. These novel findings suggest that although replacing SB with PA may help enhance sleep quality in pregnant women, particularly during the first trimester, the underlying associations are likely multifaceted. The variable relationships observed emphasize the importance of considering patterns and domains of SB and intensity of PA used as a replacement, rather than total duration solely, to improve prenatal sleep quality, especially during early pregnancy.

## Figures and Tables

**Table 1:** Participants’ demographics and health-related measures (n = 935).

Measure	Mean ± SD, n (%)
Age (years old)	30.0 ± 5.6
Height (cm)	158.7 ± 6.4
Weight (kg)	69.1 ± 14.7
Term of Pregnancy	
First Trimester	225 (24.1%)
Second Trimester	317 (33.9%)
Third Trimester	393 (42.0%)
Education	
Postgraduate	29 (3.1%)
Undergraduate	520 (55.6%)
Diploma or Less	386 (41.3%)
Occupation	
Student	56 (6.0%)
Public Sector Employee	68 (7.3%)
Private Sector Employee	94 (9.9%)
Housewife	718 (76.8%)
Currently Smoking	
Yes	21 (2.2%)
No	914 (97.8%)
Have Chronic Disease	
Yes	82 (8.8%)
No	853 (91.2%)
Have Children	
Yes	579 (61.9%)
No	356 (38.1%)
Total SB (hours/day)	6.8 ± 3.5
Leisure SB (hours/day)	5.1 ± 2.9
Occupational SB (hours/day)	0.5 ± 1.2
Commuting SB (hours/day)	1.2 ± 1.0
MPA (min/day)	30.0 ± 46.6
VPA (min/day)	2.1 ± 9.1
MVPA (min/day)	32.1 ± 48.2
Global PSQI	7.7 ± 3.7

cm: centimeter, kg: kilogram, n: number, min: minutes, MPA: moderate physical activity, MVPA: moderate-to-vigorous physical activity, SB: sedentary behavior, SD: standard deviation, PSQI: Pittsburgh Sleep Quality Index, VPA: vigorous physical activity.

**Table 2:** Influence of substituting total SB with PA on sleep quality in pregnant women.

Variables	Overall Sample (n = 935)	First Trimester (n = 225)	Second Trimester (n = 317)	Third Trimester (n = 393)
	B ± SE (*p*-Value)	B ± SE (*p*-Value)	B ± SE (*p*-Value)	B ± SE (*p*-Value)
Replacing total SB per day with MPA (30 min/day)	−0.064 ± 0.079 0.419	−0.476 ± 0.243 0.051	−0.090 ± 0.132 0.496	−0.070 ± 0.109 0.519
Replacing total SB per day with VPA (30 min/day)	**2.473 ± 0.398** **<0.001**	**6.875 ± 1.365** **<0.001**	**4.286 ± 0.831** **<0.001**	**1.390 ± 0.467** **0.003**
Replacing total SB per day with MVPA (30 min/day)	0.070 ± 0.078 0.368	−0.121 ± 0.247 0.626	0.139 ± 0.129 0.284	0.011 ± 0.107 0.919
Replacing total SB on a weekday with MPA (30 min/day)	−0.078 ± 0.083 0.325	**−0.519 ± 0.248** **0.038**	−0.124 ± 0.142 0.383	−0.053 ± 0.113 0.638
Replacing total SB on a weekday with VPA (30 min/day)	**2.459 ± 0.399** **<0.001**	**6.758 ± 1.373** **<0.001**	**4.296 ± 0.832** **<0.001**	**1.422 ± 0.471** **0.003**
Replacing total SB on a weekday with MVPA (30 min/day)	0.056 ± 0.082 0.496	−0.184 ± 0.254 0.469	0.126 ± 0.139 0.364	0.023 ± 0.112 0.836
Replacing total SB on a weekend day with MPA (30 min/day)	−0.024 ± 0.109 0.824	−0.333 ± 0.297 0.263	−0.012 ± 0.178 0.944	−0.135 ± 0.160 0.401
Replacing total SB on a weekend day with VPA(30 min/day)	**2.512 ± 0.405** **<0.001**	**6.944 ± 1.369** **<0.001**	**4.408 ± 0.852** **<0.001**	**1.340 ± 0.476** **0.005**
Replacing total SB on a weekend day with MVPA (30 min/day)	0.112 ± 0.109 0.305	0.072 ± 0.303 0.812	0.170 ± 0.181 0.349	−0.033 ± 0.159 0.836

All models were adjusted for age, smoking status, having children, education, occupation, and chronic disease status. B: beta coefficient, MPA: moderate physical activity, MVPA: moderate-to-vigorous physical activity, SB: sedentary behavior, SE: standard error, VPA: vigorous physical activity. Bold indicates significant association (*p* < 0.05).

**Table 3:** Influence of substituting leisure SB with PA on sleep quality in pregnant women.

Outcomes	Overall Sample (n = 935)	First Trimester (n = 225)	Second Trimester (n = 317)	Third Trimester (n = 393)
	B ± SE (*p*-Value)	B ± SE (*p*-Value)	B ± SE (*p*-Value)	B ± SE (*p*-Value)
Replacing leisure SB per day with MPA (30 min/day)	−0.058 ± 0.081 0.475	−0.485 ± 0.252 0.055	−0.069 ± 0.134 0.608	−0.065 ± 0.112 0.558
Replacing leisure SB per day with VPA (30 min/day)	**2.463 ± 0.399** **<0.001**	**6.875 ± 1.372** **<0.001**	**4.327 ± 0.838** **<0.001**	**1.378 ± 0.468** **0.003**
Replacing leisure SB per day with MVPA (30 min/day)	0.078 ± 0.079 0.326	−0.130 ± 0.257 0.615	0.151 ± 0.132 0.254	0.020 ± 0.109 0.858
Replacing leisure SB on a weekday with MPA (30 min/day)	−0.104 ± 0.087 0.232	**−0.590 ± 0.258** **0.023**	−0.163 ± 0.147 0.268	−0.042 ± 0.120 0.725
Replacing leisure SB on a weekday with VPA (30 min/day)	**2.413 ± 0.400** **<0.001**	**6.714 ± 1.369** **<0.001**	**4.313 ± 0.836** **<0.001**	**1.420 ± 0.474** **0.003**
Replacing leisure SB on a weekday with MVPA (30 min/day)	0.030 ± 0.086 0.727	−0.245 ± 0.264 0.355	0.078 ± 0.145 0.590	0.035 ± 0.118 0.768
Replacing leisure SB on a weekend day with MPA (30 min/day)	0.081 ± 0.125 0.520	−0.087 ± 0.342 0.800	0.157 ± 0.198 0.428	−0.150 ± 0.188 0.427
Replacing leisure SB on a weekend day with VPA (30 min/day)	**2.597 ± 0.409** **<0.001**	**7.217 ± 1.380** **<0.001**	**4.633 ± 0.859** **<0.001**	**1.313 ± 0.482** **0.007**
Replacing leisure SB on a weekend day with MVPA (30 min/day)	0.220 ± 0.126 0.080	0.295 ± 0.354 0.406	0.335 ± 0.203 0.100	−0.032 ± 0.186 0.863

All models were adjusted for age, smoking status, having children, education, occupation, and chronic disease status, and simultaneously adjusted for other domain-specific SB. B: beta coefficient, MPA: moderate physical activity, MVPA: moderate-to-vigorous physical activity, SB: sedentary behavior, SE: standard error, VPA: vigorous physical activity. Bold indicates significant association (*p* < 0.05).

**Table 4:** Influence of substituting times spent in domain-specific SB on sleep quality in pregnant women.

Outcomes	Overall Sample (n = 935)	First Trimester (n = 225)	Second Trimester (n = 317)	Third Trimester (n = 393)
	B ± SE (*p*-Value)	B ± SE (*p*-Value)	B ± SE (*p*-Value)	B ± SE (*p*-Value)
Replacing leisure SB per day with occupational SB per day (30 min/day)	−0.045 ± 0.055 0.410	−0.058 ± 0.121 0.631	0.019 ± 0.094 0.838	−0.083 ± 0.081 0.305
Replacing leisure SB per day with commuting SB per day (30 min/day)	0.044 ± 0.069 0.523	−0.080 ± 0.142 0.576	0.134 ± 0.115 0.245	0.050 ± 0.105 0.635
Replacing occupational SB per day with commuting SB per day (30 min/day)	0.089 ± 0.084 0.291	−0.022 ± 0.187 0.907	0.115 ± 0.141 0.418	0.133 ± 0.125 0.288

All were models adjusted for MPA, VPA, age, smoking status, having children, education, occupation, and chronic disease status, and simultaneously adjusted for other domain-specific SB. B: beta coefficient, SB: sedentary behavior, SE: standard error.

## Data Availability

The raw data supporting the conclusions of this article will be made available by the authors on request.

## Supplementary Materials

The following supporting information can be downloaded at: https://www.mdpi.com/article/10.3390/healthcare13192397/s1, Table S1: Participants’ demographics and health-related measures by trimester, Table S2: Influence of substituting occupational SB with PA on sleep quality in pregnant women, Table S3: Influence of substituting commuting SB with PA on sleep quality in pregnant women.
