# Supplementary material for: Isotemporal Associations of Patterns and Domains of Sedentary Behavior and Physical Activity with Sleep Quality in Pregnant Women in Saudi Arabia
Source: Healthcare (Basel). 2025 Sep 23;13(19):2397. doi: 10.3390/healthcare13192397 (PMC12523707; doi:10.3390/healthcare13192397)
Supplement: Supplementary file 1 [file healthcare-13-02397-s001.zip › healthcare-3810553-supplementary.pdf]

Table S1. Participants' demographics and health-related measures by trimester.

Measure	First Trimester (n = 225)	Second Trimester (n = 317)	Third Trimester (n = 393)
	Mean ± SD, n (%)		
Age (years old)	29.4 ± 6.4	29.9 ± 5.6	30.4 ± 5.2
Height (cm)	158.2 ± 6.7	159.3 ± 6.4	158.5 ± 6.2
Weight (kg)	64.6 ± 12.7	67.3 ± 13.0	73.2 ± 15.9
Education			
Postgraduate	10 (4.4%)	10 (3.2%)	9 (2.3%)
Undergraduate	125 (55.6%)	190 (59.9%)	205 (52.2%)
Diploma or Less	90 (40.0%)	117 (36.9%)	179 (45.5%)
Occupation			
Student	23 (10.2%)	19 (6.0%)	14 (3.6%)
Public Sector Employee	18 (8.0%)	23 (7.3%)	27 (6.9%)
Private Sector Employee	24 (10.7%)	27 (8.5%)	42 (10.7%)
Housewife	160 (71.1%)	248 (78.2%)	310 (78.9%)
Currently Smoking			
Yes	7 (3.1%)	10 (3.2%)	4 (1.0%)
No	218 (96.9%)	307 (96.8%)	389 (99.0%)
Have a Chronic Disease			
Yes	22 (9.8%)	21 (6.6%)	39 (9.9%)
No	203 (90.2%)	296 (93.4%)	354 (90.1%)
Have Children			
Yes	128 (56.9%)	178 (56.2%)	273 (69.5%)
No	97 (43.1%)	139 (43.8%)	120 (30.5%)
Total SB (hours/day)	6.7 ± 3.2	6.8 ± 3.7	6.7 ± 3.5
Leisure SB (hours/day)	5.3 ± 2.9	5.1 ± 3.0	5.0 ± 2.9
Occupational SB (hours/day)	0.4 ± 1.0	0.5 ± 1.2	0.5 ± 1.2
Commuting SB (hours/day)	1.1 ± 1.0	1.2 ± 1.1	1.2 ± 1.0
MVPA (min/day)	24.5 ± 32.8	34.8 ± 51.5	34.3 ± 52.4
MPA (min/day)	22.5 ± 31.5	32.4 ± 49.1	32.5 ± 51.1
VPA (min/day)	2.0 ± 5.6	2.4 ± 7.9	1.9 ± 11.4
Global PSQI	7.9 ± 3.9	7.9 ± 3.8	7.4 ± 3.5

cm: centimeter, kg: kilogram, n: number, min: minutes, MPA: moderate physical activity, MVPA: moderate-to-vigorous physical activity, SB: sedentary behavior, SD: standard deviation, PSQI: Pittsburgh Sleep Quality Index, VPA: vigorous physical activity

Table S2: Influence of substituting occupational SB with PA on sleep quality in pregnant women.

Outcomes	Overall Sample (n = 935)	First Trimester (n = 225)	Second Trimester (n = 317)	Third Trimester (n = 393)
	B ± SE (*p*-value)	B ± SE (*p*-value)	B ± SE (*p*-value)	B ± SE (*p*-value)
Replacing occupational SB per day with MPA (30 min/day)	-0.013 ± 0.095<br>0.895	-0.428 ± 0.295<br>0.149	-0.088 ± 0.155<br>0.570	0.018 ± 0.133<br>0.894
Replacing occupational SB per day with VPA (30 min/day)	**2.508 ± 0.401**<br>**<0.001**	**6.933 ± 1.381**<br>**<0.001**	**4.308 ± 0.832**<br>**<0.001**	**1.461 ± 0.474**<br>**0.002**
Replacing occupational SB per day with MVPA (30 min/day)	0.130 ± 0.094<br>0.168	-0.076 ± 0.305<br>0.802	0.179 ± 0.151<br>0.239	0.100 ± 0.131<br>0.445
Replacing occupational SB on a weekday with MPA (30 min/day)	0.023 ± 0.101<br>0.822	-0.370 ± 0.299<br>0.218	0.003 ± 0.170<br>0.985	0.016 ± 0.140<br>0.909
Replacing occupational SB on a weekday with VPA (30 min/day)	**2.540 ± 0.402**<br>**<0.001**	**7.131 ± 1.391**<br>**<0.001**	**4.367 ± 0.832**<br>**<0.001**	**1.460 ± 0.476**<br>**0.002**
Replacing occupational SB on a weekday with MVPA (30 min/day)	0.167 ± 0.100<br>0.097	-0.038 ± 0.311<br>0.904	0.278 ± 0.168<br>0.098	0.100 ± 0.138<br>0.469
Replacing occupational SB on a weekend day with MPA (30 min/day)	-0.322 ± 0.313<br>0.302	-1.202 ± 0.736<br>0.104	-0.747 ± 0.537<br>0.165	0.035 ± 0.467<br>0.940
Replacing occupational SB on a weekend day with VPA (30 min/day)	**2.195 ± 0.501**<br>**<0.001**	**6.299 ± 1.487**<br>**<0.001**	**3.616 ± 0.990**<br>**<0.001**	**1.479 ± 0.657**<br>**0.025**
Replacing occupational SB on a weekend day with MVPA (30 min/day)	-0.194 ± 0.318<br>0.541	-0.543 ± 0.770<br>0.482	-0.557 ± 0.556<br>0.318	0.101 ± 0.472<br>0.830

All models were adjusted for age, smoking status, having children, education, occupation, and chronic disease status, and simultaneously adjusted for other domain-specific SB. B: beta coefficient, MPA: moderate physical activity, MVPA: moderate-to-vigorous physical activity, SB: sedentary behavior, SE: standard error, VPA: vigorous physical activity. Bold indicates significant association ($p<0.05$).

Table S3: Influence of substituting commuting SB with PA on sleep quality in pregnant women.

Outcomes	Overall Sample (n = 935)	First Trimester (n = 225)	Second Trimester (n = 317)	Third Trimester (n = 393)
	B ± SE (*p*-value)	B ± SE (*p*-value)	B ± SE (*p*-value)	B ± SE (*p*-value)
Replacing commuting SB per day with MPA (30 min/day)	-0.102 ± 0.097<br>0.293	-0.406 ± 0.264<br>0.126	-0.203 ± 0.165<br>0.221	-0.115 ± 0.136<br>0.398
Replacing commuting SB per day with VPA (30 min/day)	**2.419 ± 0.404**<br>**<0.001**	**6.955 ± 1.375**<br>**<0.001**	**4.193 ± 0.842**<br>**<0.001**	**1.328 ± 0.482**<br>**0.006**
Replacing commuting SB per day with MVPA (30 min/day)	0.022 ± 0.096<br>0.823	-0.054 ± 0.270<br>0.842	0.024 ± 0.165<br>0.886	-0.053 ± 0.136<br>0.695
Replacing commuting SB on a weekday with MPA (30 min/day)	-0.093 ± 0.121<br>0.440	-0.378 ± 0.330<br>0.253	-0.154 ± 0.209<br>0.464	-0.154 ± 0.166<br>0.355
Replacing commuting SB on a weekday with VPA (30 min/day)	**2.429 ± 0.413**<br>**<0.001**	**6.993 ± 1.405**<br>**<0.001**	**4.240 ± 0.852**<br>**<0.001**	**1.258 ± 0.494**<br>**0.010**
Replacing commuting SB on a weekday with MVPA (30 min/day)	0.019 ± 0.122<br>0.876	-0.083 ± 0.344<br>0.811	0.077 ± 0.212<br>0.716	-0.099 ± 0.167<br>0.551
Replacing commuting SB on a weekend day with MPA (30 min/day)	-0.125 ± 0.224<br>0.577	-0.473 ± 0.551<br>0.392	-0.330 ± 0.373<br>0.376	0.004 ± 0.326<br>0.990
Replacing commuting SB on a weekend day with VPA (30 min/day)	**2.397 ± 0.448**<br>**<0.001**	**6.899 ± 1.437**<br>**<0.001**	**4.063 ± 0.909**<br>**<0.001**	**1.443 ± 0.559**<br>**0.010**
Replacing commuting SB on a weekend day with MVPA (30 min/day)	0.029 ± 0.227<br>0.900	0.015 ± 0.576<br>0.979	-0.116 ± 0.385<br>0.763	0.090 ± 0.328<br>0.784

All models were adjusted for age, smoking status, having children, education, occupation, and chronic disease status, and simultaneously adjusted for other domain-specific SB. B: beta coefficient, MPA: moderate physical activity, MVPA: moderate-to-vigorous physical activity, SB: sedentary behavior, SE: standard error, VPA: vigorous physical activity. Bold indicates significant association (p<0.05).
